# The Medical Uses of Electricity in the Treatment of Nervous Affections

**Published:** 1860-06

**Authors:** 


THE MEDICAL USES OF ELECTRICITY IN THE TREATMENT OF NERVOUS AFFECTIONS: A new and important medical work, just issued by Messrs. TICKNOR & FIELDS.

This is a thoroughly systematic work of over 700 pages, and finely illustrated with nearly 100 cuts, showing not only the best "*Methods*" for the therapeutical employment of Electricity in the various nervous diseases, but also *showing the anatomy of the parts* (nerve-trunks and muscle-fibres) liable to be involved; moreover presenting a concise view and means of diagnosis of the great variety of nervous affections met with in every-day practice. This work is from the pen of ALFRED C. GARRATT, M. D., of Boston, who of late years, it is well known, has made this difficult department of medicine his *specialty.* It is addressed to medical students, and is dedicated to Dr. JOHN HOMANS, President of the Massachusetts Medical Society. It is intended for the professional eye. There is no similar work in the English language.

A more extended notice will be given after we have had time to examine the work more carefully.

